# Editorial: Therapeutic Opportunities and Innovative Biomarkers in Tumor Microenvironment

**DOI:** 10.3389/fonc.2021.803414

**Published:** 2021-11-30

**Authors:** Kexin Xu, Farah Rahmatpanah, Zhenyu Jia

**Affiliations:** ^1^ Department of Molecular Medicine, University of Texas Health Science Center at San Antonio, San Antonio, TX, United States; ^2^ Department of Pathology and Laboratory Medicine, University of California, Irvine, Irvine, CA, United States; ^3^ Department of Botany and Plant Sciences, University of California, Riverside, Riverside, CA, United States

**Keywords:** therapeutic targets, biomarkers, diagnosis, prognosis, methodology, tumor microenvironment

The past few years have witnessed an explosion of cancer immunology research. Nowadays, it has been well accepted that cancer cells are surrounded and infiltrated by tumor stroma, termed the tumor microenvironment (TME). Alongside cancer cells, TME consists of a heterogenous collection of nontransformed cells, including immune cells, endothelial cells and fibroblasts (1). It is also composed of various non-cellular components, such as the structural matrix, secreted macromolecules, and extracellular vesicles (2). The dynamic, constant and mutual communications between cancer cells and stromal factors contribute significantly to tumorigenesis and cancer progression (3). On one hand, the malignant cells invade healthy tissues and reshape the overall structure of surrounding milieu to induce peripheral immune tolerance, support tumor angiogenesis and disrupt stroma integrity (4). They do so by altering the expression of genes important for the functions of extracellular matrix (ECM) through genetic or epigenetic aberrations, secreting a significant amount of tumor-derived circulating materials, and activate or suppress complex signaling networks to hijack the non-neoplastic cells for their own benefits. On the other hand, tumor-adjacent non-transformed cells are recruited by cancer cells from local host stroma and penetrate into tumors to assist in cancer initiation, growth, invasion, metastasis and responses to therapies (5). These tumor-associated stromal cells (TASCs) support tumorigenesis and cancer progression through direct cell-to-cell contacts or secreting many pro-tumorigenic factors, which provide metabolites, energy, growth signals, metastatic and angiogenesis cues for understanding the cancer cells. In addition, ECM provides a biochemical and biomechanical framework within which cancer cells prosper, and therefore the acellular components within ECM can determine the physical properties, composition and topography of cancer cells, which play an important role in the speed of cancer migration (6).

Intimate and reciprocal communications between cancer cells and surrounding environment inspire the idea that cancer can potentially be treated by targeting the infiltrated nonmalignant components. Moreover, it has been demonstrated that tumor stroma promotes resistance of cancer cells to many anticancer therapies that focus on cancer cells specifically (7). Therefore, development of anticancer agents and identification of cancer biomarkers now have been switched from cancer-centric to TME-centric. Considering the diverse compositions, TME provides a vital new source for the discovery of anticancer drug targets with context-dependent functions in cancer (8). Additionally, in several preclinical and clinical studies, the stromal factors demonstrate significant diagnostic and prognostic values, indicating the potential use of such substances as tumor biomarkers besides many currently used ones that are expressed by cancer cells [(9); Petersen et al.]. In the last decades, new analytical approaches, technologies, biological models and conceptual breakthroughs emerged, which make it possible to identify novel TME-associated drug targets and biomarkers and to explore the mechanisms of their actions. All these advances drive the application of multiple FDA-approved immunotherapies and corresponding predictive biomarkers in clinical practice for the treatment of various types of hematopoietic cancers and solid tumors (10, 11).

## Potential Diagnostic, Prognostic, and Therapeutic Biomarkers in Tumor Microenvironment

Biomarkers based on the cellular and non-cellular components of TME have proven clinical values. They serve as indicators of cancer pathogenesis and predict responses to a variety of cancer treatment options including immunotherapy. A number of studies support that infiltration of untransformed cells, such as macrophages (Niu et al.), lymphocytes (Dai et al.; Liu et al.; Fu et al.), and cancer-associated fibroblasts (CAFs) (12), provides important diagnostic and prognostic information for cancer patients. The density levels of tumor-infiltrating lymphocytes (TILs) are shown to link with survival rates in a variety of cancers (13–15), and therefore quantifying TILs has been devised to assess therapeutic prediction. In one study, the content of twenty-two types of TILs in tumors from patients with head and neck squamous cell carcinoma (HNSCC) was analyzed and compared to that in adjacent normal tissues based on the gene expression and clinical data in TCGA database (Liu et al.). Some types of TILs showed notable differences between normal and cancerous tissues. Furthermore, survival analysis showed that patients with more naïve B cells and regulatory T cells tend to live longer, whereas the lifespans of those with more eosinophils and activated mast cells are shorter. Together, these analyses suggest that presence of certain TILs in the TME of HNSCC tumors may provide important diagnostic and prognostic information. In another study, the infiltration degree of CD3+, CD4+ and CD8+ T cells in tumors from patients with high-grade serous ovarian cancer (HGSOC) was significantly stronger than that in the normal counterparts (Dai et al.). In addition, higher densities of CD3+ and CD8+ TILs in (HGSOC) were found to closely associate with higher levels of lactate dehydrogenase (LDH), which is a poor prognostic biomarker for many types of solid cancer (16, 17).

Besides the densities of infiltrated non-tumorigenic cells, expression of genes playing roles in regulation of TME may also have great diagnostic and prognostic values in certain types of human cancer [Zhou et al.; Fu et al. (18, 19)]. For instance, levels of four genes functionally involved in immune signaling (*IFI16*, *LMCB1*, *RHBDF2* and *TACC3*) were found upregulated in clear cell renal cell carcinoma, which were associated with poor prognosis (Lv et al.). Immune-related non-coding RNAs (ncRNAs) have also been advocated as powerful prognostic indicators (Wang et al.; Cheng et al.; Guo et al.). In one study, the relationship between expression of a unique class of ncRNAs called circular RNAs (circRNAs) and biochemical recurrence (BCR) in patients with prostate cancer was analyzed (Wang et al.). In another study, an intracellular competitive endogenous RNA (ceRNA) network, the RNA molecules that share the microRNA (miRNA) recognition elements with the target messenger RNAs (mRNAs) and therefore compete for binding to miRNAs (20), was identified in acute myeloid leukemia (AML), which showed differential expression patterns between samples with low and high immune cell infiltration scores (Cheng et al.).

Recent studies show that molecular compositions and biomechanical properties of ECM can both be used as valuable diagnostic tools and to foresee favorable or unfavorable outcome of a certain type of cancer (Petersen et al.). For example, the circulating ECM-related proteins, collagens, were found significantly upregulated in plasma samples from women with breast carcinoma compared to normal specimens, and combination of the collagen amounts can potentially be informative in discriminating breast cancer patients from those with benign disease (21). In addition, it was reported that increased rigidity of ECM promotes resistance to chemotherapy in pancreatic ductal adenocarcinoma and the matrix stiffness exhibited prognostic power in response specifically to paclitaxel (22). Exosome, one of the membrane-bound extracellular vesicles in ECM, assists in the communications between cancer cells and normal tissues or among different components within tumor stroma (23). One study showed that exosomes enhanced the metastasis of lung cancer cells to brain (Gan et al.). They did so by inducing the brain endothelial cells to release DKK-1 protein, an inhibitor of the canonical Wnt/β-catenin pathway that modulates microenvironment to facilitate cancer metastasis (24), and enhanced colonization of cancer cells into brain. It has also been suggested that expression of exosomal circRNAs can be used as clinical tumor biomarkers for early diagnosis, tumor prognosis and prediction of postoperative recurrence (Xu et al.).

## Immunotherapeutic Targets and Predictive Biomarkers for Cancer Immunotherapy

Immunotherapy, which deploys the immune system to treat human diseases, has revolutionized cancer treatment. Currently, there are five different classes of immunotherapies that have been integrated into the standard treatment guidelines for cancer patients, i.e., cell-based immunotherapies such as chimeric antigen receptor (CAR) T cells, immunomodulators like cytokine INF-α and immune checkpoint inhibitors (ICIs), cancer vaccines, antibody-based anticancer agents and at last oncolytic viruses (25). Although immunotherapy produces more durable responses and fewer side effects, these therapies confer benefits in only a select group of cancer types and usually in a minority of patients with those cancers (26, 27). Primary and acquired resistance to immunotherapy urge the identification of alternative therapeutic options to potentiate immune surveillance and biomarkers that link patient subpopulations to immunotherapy efficacy.

It has been demonstrated that the tumoricidal activity of some immune cells, like T cells, are profoundly affected by the peptide epitopes that emerge at the surface of cancer cells. Such epitopes may be originated from cancer neoantigens, the immunogenic products generated by somatic mutations in tumor DNA. Thus, neoantigens are specifically expressed on the surface of neoplastic but not normal cells. Due to these features, neoantigens are not expected to induce autoimmune toxicity and therapeutic approaches based on neoantigens sound highly attractive (Han et al.). Another repertoire of tumor-associated epitopes is the antigens encoded by viral open reading frames in virus-infected cancer cells. In one study, gene expression profiling in tissue samples from breast cancer patients with HIV showed upregulated transcripts of human endogenous retroviruses (HERV) compared to HIV-negative samples, which is associated with an increase in the expression of nearby oncogenes in host cells, an enrichment of extracellular matrix organization and a higher concentration of tumor-infiltrating lymphocytes (Curty et al.).

In addition to the peptide epitopes presented on the cancer cell surface, many proteins are also reported to exert specific biological functions within the tumor microenvironment, serving as potential immunotherapeutic targets (Xie et al.; Zhu et al.; (28)). For example, Ser/Arg-rich splicing factor (SRSF3) was demonstrated to bind at 3’-UTRs of some innate immune genes and thus regulate the protein synthesis, indicating a role of SRSF3-mediated translational regulation in innate immunity (28). As another example, TFEB, a member of the Microphthalmia family of bHLH-LZ transcription factors (MiT/TFE), was shown to activate the transcription of genes encoding matrix metalloproteinases, such as MMP2 and MMP9, and those functioning in lysosome biogenesis like ABCA2, which results in the degradation of the extracellular matrix and subsequently enhances the migration and invasion of prostate cancer cells (Zhu et al.). These studies offer new potential drug targets that will expand the reservoirs of cancer immunotherapy agents.

Many studies have also been carried out to recognize predictive biomarkers that link with the efficacy of immunotherapy, particularly with the immune checkpoint inhibitors (ICIs). Ferroptosis, a specific type of programmed cell death due to accumulation of excessive iron and unchecked lipid peroxidation (29), has been suggested to contribute to the anti-tumor effects of immunotherapy (30). One study constructed a Comprehensive Index of Ferroptosis and Immune status (CIFI) in hepatocellular carcinoma (HCC), based on the expression of twenty-seven prognostic ferroptosis- and immune-related genes signatures (Liu et al.). HCC patients in the CIFI-high subgroup had significantly higher frequency of TP53 mutation and higher levels of tumor heterogeneity, associated with worse survival rates and immunotherapy failure. In head and neck squamous cell carcinoma (HNSCC), integrative analysis of the clinical information and gene mutation data suggest that HNSCC patients, who are at least 65-years old and carry wild-type *TP53* gene but mutant *PIK3CA* and *ARID1A* genes, showed favorable responses to ICI treatment and prolonged overall survival rate (Zhang et al.). Finally, five immune-related genes (*IFIH1*, *CTSG*, *STC2*, *SECTM1* and *BIRC5*) were selected to build an immune gene-related prognostic model (IGRPM), which accurately predicts longer survival time and better responses to immunotherapy in the low-risk subpopulation of patients with soft tissue sarcoma (STS) (Gu et al.). All the information provided in these studies help guide treatment selection for a particular type of cancer and stratify patients who are most likely to benefit from immunotherapy.

## Innovative Biological Models or Experimental Platforms for Investigating TME

For the past few years, there has been a tremendous progress in the development of methodologies and *ex vivo* models to study the biological characteristics of TME. These tools not only enable the accurate assessment of the effectiveness of new drug targets in a milieu resembling the original tumors, but also provide valuable and systematical ways for biomarker identification, bridging the translational gap between pre-clinical and clinical settings.

Mass cytometry, or CyTOF, has been one of the technologies that are commonly used to analyze the phenotypic and functional attributes of distinct immune cells at the single-cell level (31). High throughput CyTOF methodology enables high-dimensional and unbiased examination of immune system and simultaneous interrogation of a large number of parameters that will be able to provide spatial and cell-cell interaction information (Fu et al.).

Recent advances on three-dimensional (3D) *in vitro* and *ex vivo* cell culture models, especially those co-cultured with the ECM, open doors for biomarker identification and immunotherapy evaluation (Petersen et al.). These models mimic the compositional and mechanical properties of TME and closely recapitulate the interactions between cancer cells and stromal components. Current 3D culture systems include tumor spheroids, scaffold-based tumor models and cancer cells grown in the 3D printed construct, etc., which leverage the engineered tumor microenvironment to monitor the malignant phenotypes (32). In addition, matrix manipulation, such as use of synthetic and natural gel or nano- and micropatterning, renders a more natural behavior in cancer cells (33). Compared to the classical two-dimensional culture of cancer cells alone, 3D models co-cultured with ECM better reflect the responses of cancer cells to a particular treatment option in clinical scenario and more reliably justify the diagnostic or prognostic power of a biomarker.

Discovery of the Clustered Regularly Interspaced Short Palindromic Repeat (CRISPR)-associated protein 9 (CRISPR-Cas9) system, one of the most efficient and versatile genome-editing systems that enables site-specific changes of single or multiple target genes (34), has revolutionized cancer research including cancer immunology (35). For example, genome-wide CRISPR-Cas9 screening pinpointed RAD9A, a cell cycle checkpoint protein, that upregulates the infiltrating levels of regulatory T cells and consequently confers resistance of prostate cancer cells to the anticancer drug metformin (Chen et al.). As such, CRISPR-Cas9 is advantageous due to its accuracy, efficiency and specificity, and therefore enables the mounting emergence of TME-related drug targets or biomarkers.

## Current Limitations and Future Implications

Targeting TME has paved a wider path for human cancer intervention, which will likely fulfill the personalized immunotherapy in properly selected cancer patients to maximize a clinical benefit. Despite an increase in our knowledge of TME, lots of questions remain unaddressed regarding the origins, functions and genetic alterations of distinct components in tumor stroma. Major clinical challenges still need to be resolved, particularly in how to identify the cancer patients who may respond to a particular immunotherapeutic agent and how to establish an optimal scheme so that the biomarkers can be reliably used to track cancer development and progression or predict responses to immunotherapy.

All the biomarkers and therapeutic targets discussed here are novel discoveries in cancer research; however, they are currently at an early stage in terms of their clinical utility. It is not clear in what setting they can be applied to which type of cancer and additional evaluation is needed before integration into routine medical practice. A better understanding of the detailed mechanisms underlying the complex interactions between cancer and the immune system will accelerate the development of clinically useful biomarkers and improved treatment options for patients. Compared to targeting single factor, optimization of a combined use of immune-based therapies or biomarkers in future endeavor will bring more benefits to cancer patients. Furthermore, identification of other TME-associated components, such as circulating tumor cells and cell-free DNA in the blood stream, can extend the clinical scenarios where the candidate immunotherapeutic targets or biomarkers may be applied. Future investigation is warranted to detect valuable biomarkers of low concentrations in non-invasive liquid biopsy.

Characterizing TME in the context of tumors and the functions of stromal components in a systematical manner is the major focus in the development of experimental methods and models for cancer studies. It is essential to establish pre-clinical settings that contain all the hallmarks of human immunity, represent organ-specific tumor microenvironment and maximize the personalized immunotherapy through composite biomarkers. Further multi-omics, biochemical, and animal studies are necessary to ascertain the roles of TME-derived factors as causative and mechanistic biomarkers or alternative drug targets.

## Author Contributions

All authors listed have made a substantial, direct, and intellectual contribution to the work and approved it for publication.

## Funding

This work was supported by the UC Cancer Research Coordinating Committee Competition Award to ZJ.Grant number (NIH/NCI R01CA226570 (PI: Rahmatpanah).

## Conflict of Interest

The authors declare that the research was conducted in the absence of any commercial or financial relationships that could be construed as a potential conflict of interest.

## Publisher’s Note

All claims expressed in this article are solely those of the authors and do not necessarily represent those of their affiliated organizations, or those of the publisher, the editors and the reviewers. Any product that may be evaluated in this article, or claim that may be made by its manufacturer, is not guaranteed or endorsed by the publisher.
